# Antiosteoporosis Effect and Possible Mechanisms of the Ingredients of Fructus Psoraleae in Animal Models of Osteoporosis: A Preclinical Systematic Review and Meta-Analysis

**DOI:** 10.1155/2021/2098820

**Published:** 2021-11-24

**Authors:** Zhou Lin, Junju Zheng, Jiaru Chen, Mangmang Chen, Shuangxia Dong

**Affiliations:** ^1^Department of Orthopaedic Surgery, Wenzhou Central Hospital, Wenzhou, Zhejiang 325000, China; ^2^Department of Respiratory Medicine, Wenzhou Central Hospital, Wenzhou, Zhejiang 325000, China

## Abstract

**Objective:**

*Fructus Psoraleae* (*FP*) and its ingredients (IFP) have a variety of biological activities and are widely used to treat osteoporosis (OP). Herein, we conducted a systematic review to evaluate the efficacy of IFP for an animal model of OP from the current literatures. Potential mechanisms of IFP in the treatment of OP were also summarized.

**Materials and Methods:**

We carried out a search for electronic literature in the PubMed, Chinese National Knowledge Infrastructure, EMBASE, Wanfang, Web of Science, Chinese Biomedical Literature Database, and Cochrane Library, as well as Chinese VIP databases targeting articles published from inception to June 2021. The inclusion criteria were animal studies that assessed the efficacy and safety of IFP for OP, regardless of publication status or language. The exclusion criteria included (1) other types of studies (in vitro studies, case reports, clinical trials, reviews, abstracts, comments, and editorials), (2) combination with other compounds, (3) compared with other traditional Chinese medicine, (4) not osteoporosis or bone loss model, (5) studies with insufficient data, (6) lack of a control group, and (7) duplicate publications. The modified Collaborative Approach to Meta-Analysis and Review of Animal Data from Experimental Stroke (CAMARADES) 10-item quality checklist was used to evaluate the risk of bias of included studies. We computed the relative risk (RR) and the standard mean difference (SMD) for dichotomous outcomes and continuous outcomes, respectively. When heterogeneity was detected or there was significant statistical heterogeneity (*P* < 0.05 or *I*^2^ > 50%), a random-effects model was employed, followed by further subgroup analysis and metaregression estimations to ascertain the origins of heterogeneity. Otherwise, we used a fixed-effects model (*P* ≥ 0.05 or *I*^2^ ≤ 50%). The primary outcome measures were bone mineral density (BMD), serum osteocalcin(S-OCN), bone volume over total volume (BV/TV), trabecular number (Tb.N), trabecular thickness (Tb.Th), trabecular separation (Tb.Sp), bone maximum load, and elasticity modulus. The secondary outcome measure was the antiosteoporosis mechanisms of IFP. The STATA 12.0 software was used to analyze the data.

**Results:**

Overall, 16 studies focusing on 379 animals were enrolled into the study. The risk of bias score of included studies ranged from 4 to 7 with an average score of 5.25. The present study provided the preliminary preclinical evidence that administration of IFP could significantly increase the S-OCN, BMD, BV/TV, and Tb.N while Tb.Th and Tb.Sp were remarkably decreased by IFP in OP model animals (*P* < 0.05). Moreover, IFP could significantly improve the bone biomechanical indicator bone maximum load and elasticity modulus (*P* < 0.05). In terms of the possible mechanisms of treatment of OP, IFP exerts anti-OP effects in animal models probably through osteoprotegerin/receptor activator of the nuclear factor-*κ*B ligand/receptor activator of nuclear factor-*κ*B (OPG/RANKL/RANK), peroxisome proliferator activated receptor *γ* (PPAR-*γ*)/Axin2/Wnt, antioxidative stress via forkhead box O3a (FoxO3a)/Axin2/Wnt, phosphatidylinositol 3-kinase/protein kinase B/mammalian target of rapamycin (PI3K/Akt/mTOR), estrogen-like effect, and gamma-aminobutyric acid/gamma-aminobutyric acid receptor (GABA/GABA_B_RI) signaling pathway.

**Conclusion:**

Taken together, the findings suggest the possibility of developing IFP as a drug or an ingredient in diet for the clinical treatment of OP. We recommend that rigorous, as well as high-quality, trials involving large sample sizes should be conducted to confirm our findings.

## 1. Introduction

Osteoporosis (OP), is a systemic skeletal disease characterized by loss of bone mass and bone microarchitectural deterioration, resulting in increased bone fragility and a greater risk of fractures, especially in the spine, hip, and wrist [1, 2]. Many risk factors are associated with OP, including age, race, smoking, alcohol, low physical activity, hormone-related factors, drugs such as glucocorticoids, low calcium, and vitamin D levels [3]. Reports have showed that about 9.9 million Americans suffer from OP, with an additional 43.1 million experiencing low bone mineral density [4]. In China, the prevalence of OP was 14.94% before 2008 and increased to 27.96% from 2012 to 2015, with the rate being higher in females relative to males [5]. Approximately, in the United States, 16% of men and 29.9% of women aged more than 50 years have OP on the basis of the diagnostic criteria of the National Bone Health Alliance [6]. With the aggravation of global population aging, OP has become a serious global health problem. OP has been attributed to decreased quality of life and increased risks of death along with an elevated burden on health systems economically [7]. Thus, the management of patients with OP is extremely urgent.

Up to now, calcium and vitamin D supplementation are the standard choices for OP treatment [8]. Pharmacological therapies, including bisphosphonates, denosumab, and teriparatide, are also recommended to reduce the risk of vertebral or hip fractures in patients with OP [9]. Estrogen therapy, menopausal estrogen plus progestogen therapy, or raloxifene is suitable for postmenopausal women [10]. However, despite the availability of numerous anti-OP medications with diverse pharmacological properties, as well as fixed-dose combination therapy, the targeted therapeutic effect is not attained in significant numbers of individuals with OP, and the mitigation of OP fracture has remained suboptimal [11, 12]. Therefore, finding a drug that is effective and safe for osteoporosis is an important challenge for the industry.

**Figure 1:**
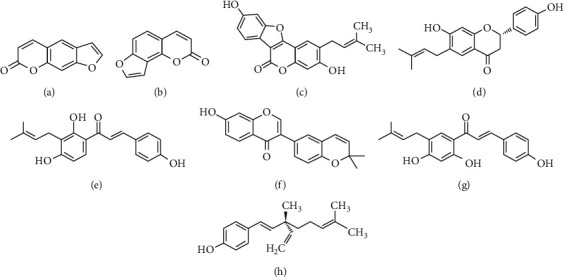
The chemical structure of IFP: (a) psoralen; (b) isopsoralen; (c) psoralidin; (d) corylifolin; (e) corylifolinin; (f) corylin; (g) bavachalcone; (h) bakuchiol.

Recently, the growing utilization of complementary and alternative medicine consists of herbal medicine in research and in clinical practice medicinal: they usually have few side effects and are easily accessible [13, 14]. Herbal medicines are typically used as a complementary and adjunct therapy for a wide range of diseases such as OP [15]. *Fructus Psoraleae* (FP) is the dried matured fruits of *Psoralea corylifolia* Linn, which has a long history and a wide range of applications in Asia, particularly in China, Japan, and Korea for their effects on OP and bone fracture [16]. Studies have shown that FP has the effects of dilating coronary artery, increasing coronary blood flow, antitumor, protecting liver, estrogen-like, and anti-OP effects [17]. The ingredients of FP (IFP) are shown in Figure 1, which mainly contains furanocoumarins (mainly psoralen and isopsoralen), coumestrol (such as psoralidin), flavonoids (mainly corylifolin, corylifolinin, corylin, and bavachalcone), and phenolic terpenoids (such as bakuchiol). Many IFP have estrogen-like effects [18]. Recent reports have shown that phytoestrogens have anti-OP effects similar to natural estrogen with less estrogen-like side effects [19]. Several studies have demonstrated that multiple IFP may possess anti-OP effects both *in vivo* and *vitro* [20, 21]. Corylifolin could prevent estrogen deficiency-induced bone loss in ovariectomized rats and induced primary human osteoblast differentiation [20]. Psoralen and bakuchiol improved osteoclast differentiation and bone resorption via inhibiting the protein kinase B (AKT) and activator protein-1 (AP-1) pathway [21]. However, the scattered evidence and uncertain mechanisms limited the application of FP and IFP in the clinic. Systematic review and meta-analysis of animal studies are considered to be a valuable tool to provide important insights into the validity of animal studies, improve the precision of estimated effects, and support further generalization to human clinical trials [22]. We speculated that IFP could exert anti-OP effects in animal models of OP. However, it is difficult to translate these beneficial effects of IFP from basic research to clinical application. Besides, the uncertain mechanisms and adverse drug reaction of IFP should also be taken into consideration, which were also the uncertainties and conflicts that underlie the hypotheticals. The evidence of studies was beneficial to find out a potential medication to prevent OP for healthy individuals and to treat OP for OP patients with less adverse drug reaction. Now clinically, FP has not been used to treat OP and there is a lack of clinical evidence, which is the focused clinical question we review and address in our study. Our study might provide a theoretical basis for the application of FP in OP. Therefore, we presented a systematic review and meta-analysis from the preclinical evidence of IFP in animal models of OP to summarize the significant outcomes on efficacy and mechanisms.

## 2. Methods

We used the PRISMA (Preferred Reporting Items for Systematic Reviews and Meta-Analyses) statement (Table 1) to perform a systematic review and meta-analysis [23]. There are no protocols preregistered for this review.

### 2.1. Database and Search Strategies

Electronic searches were performed in eight databases from their respective inception to June 2021: PubMed, EMBASE, Web of Science, Cochrane Library, Chinese National Knowledge Infrastructure, Chinese Biomedical Literature Database, Chinese VIP Database, and Wanfang Database. No language restrictions were applied. The following search terms were used in PubMed and were modified to suit other databases: “Fructus Psoraleae extract” AND “Osteoporosis.” In addition, reference lists from the resulting publications and reviews were also searched carefully for the eligible studies.

### 2.2. Eligibility Criteria

The studies were included according to the following PICOS criteria.

#### 2.2.1. Types of Participants

We included controlled studies assessing the administration of IFP for OP animal models established by different methods, regardless of animal species, age, weight, and gender.

#### 2.2.2. Types of Interventions

The treatment group received IFP as monotherapy, regardless of dosage, medicament type, route of administration, and time for the medicine application.

#### 2.2.3. Types of Controls

Blank treatment or isometric placebo was received in the control group.

#### 2.2.4. Types of Outcome Measures

The primary outcome measures were the following: (1) bone mineral density (BMD, including BMD-lumbar spine and BMD-femur), (2) serum osteocalcin(S-OCN), (3) bone volume over total volume (BV/TV), (4) trabecular number (Tb.N), (5) trabecular thickness (Tb.Th), (6) trabecular separation (Tb.Sp), (7) bone maximum load, and (8) elasticity modulus. The secondary outcome measure was the antiosteoporosis mechanisms of IFP.

#### 2.2.5. Types of Studies

Only animal studies that assessed the efficacy and safety of IFP for OP were included, regardless of publication status or language.

### 2.3. Exclusion Criteria

Exclusion criteria are as follows: (1) other types of studies (in vitro studies, case reports, clinical trials, reviews, abstracts, comments, and editorials), (2) combination with other compounds, (3) compared with other traditional Chinese medicines, (4) not osteoporosis or bone loss model, (5) studies with insufficient data, (6) lack of control group, and (7) duplicate publications.

### 2.4. Selection of Literature

We used the PRISMA flow diagram to select the included studies. The results of literature search were imported into the software Endnote X7. Two authors independently assessed the potentially eligible studies. Firstly, the titles and abstracts were screened to exclude the duplicated and apparently irrelevant ones or those that do not meet our inclusion criteria. After that, the remaining potential studies were full-text downloaded and reviewed. Any disagreement between the two above authors was sent and discussed with the third independent author.

### 2.5. Data Extraction

Two reviewers independently extracted data, and the third reviewer checked the consistency between them. A standard form was used; the extracted items included the general study information: the author's name(s), publishing date, animal species, age, gender, weight, sample size, OP modeling method, the use of anesthetics in the course of the experiment, the therapeutic regimen of the treatment and control groups, and primary and secondary outcomes and its intergroup differences. For continuous outcomes, we extracted the mean, standard deviation (SD), and participant number. The data in other forms was recalculated when possible to enable pooled analysis. If the study was involved in multiple intervention groups, we extracted data only for the group(s) involving IFP and the control group(s). Disagreements between two researchers were resolved by discussion. Whenever necessary, we contacted the authors of the studies for the missing data and additional information.

### 2.6. Quality Assessment of Included Studies

Two authors independently performed methodological quality and risk of bias assessment of the included studies using the CAMARADES 10-item quality checklist with minor modification [24]. The modification is listed as follows: D: blinded induction of model (group randomly after modeling), F: use of anesthetic without significant protective and toxic effects on bones, and G: appropriate animal model with complications or risk factors (including age, hyperlipidemia, diabetes, or hypertensive).

### 2.7. Quality Assessment of Evidence

The assessment of evidence quality was evaluated according to the GRADE criteria [25, 26]. The evidence quality of included outcomes was graded as high, moderate, low, or very low. RCTs were initially classified as having high-quality evidence. The quality of each outcome was downgraded for the following five factors: risk of bias, inconsistency, indirectness, imprecision, and publication bias. GRADE pro3.6.1 software was used for the data analysis and synthesis.

### 2.8. Statistical Analysis

The data was collected and input into the STATA software (version 12.0; StataCorp, College Station, TX) for meta-analysis. A random-effects model was applied when heterogeneity was detected or the statistical heterogeneity was high (*P* < 0.05 or *I*^2^ > 50%), and then, further subgroup study and metaregression analysis (the number of included studies was more than ten) were performed to detect the origin of heterogeneity. Otherwise, a fixed-effects model was used (*P* ≥ 0.05 or *I*^2^ ≤ 50%). To test the strength and stability of the pooled results, we performed a sensitivity analysis by omitting the individual studies one by one. Moreover, the effect of publication bias was investigated by Egger's test. Standard mean difference (SMD) was calculated for continuous outcomes.

## 3. Results

### 3.1. Study Selection

**Figure 2:**
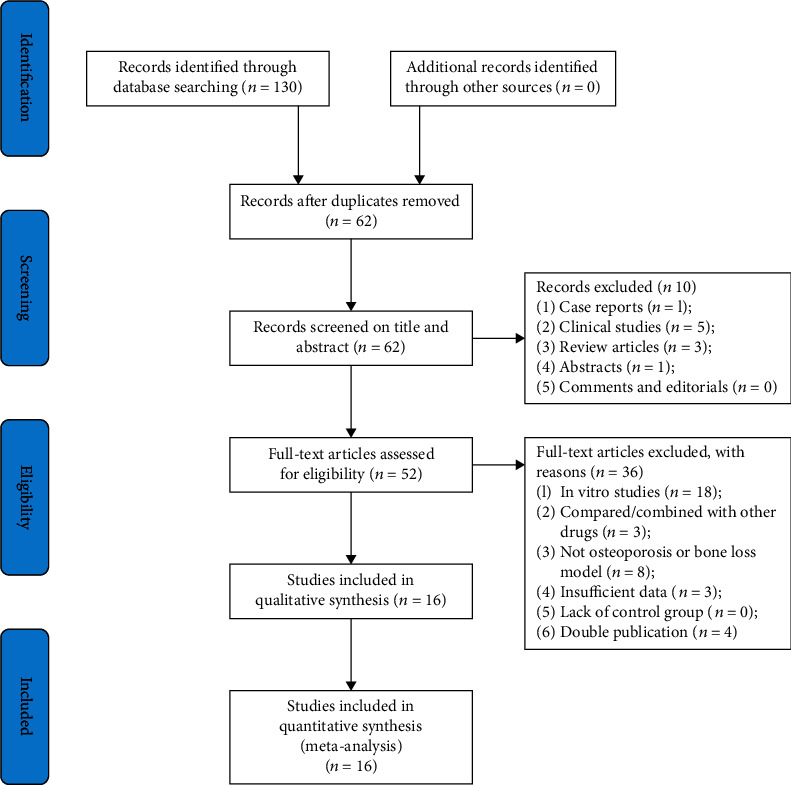
Flowchart of study selection.

We identified 130 potentially relevant hints from eight databases. Removing duplication of literature, there were 62 articles left. We excluded 10 studies that are not related to this study after reading the titles and abstracts in detail. Through reading the full text of 52 articles, 36 articles were excluded for at least one of the exclusion criteria. Finally, 16 articles [17, 27–41] were included for analysis.  Figure 2 presents the detailed flow diagram of the search and selection process.

### 3.2. General Characteristics of the Included Studies

The characteristics of all the 16 included studies are summarized and shown in Table 2. All of them were published between 2008 and 2021. All studies involved a total of 379 subjects with 191 in the experimental group vs. 188 in the control group. As for animal species, Sprague-Dawley rats were used in ten studies [27–29, 31, 32, 34, 37–39, 41], Wistar rats in three [17, 30, 40], C57BL/6 mice in two [35, 36], and ICR mice in one [33]. The weight of rats varied between 160 g and 290 g, while the weight of mice varied between 16.6 g and 22 g. Eleven studies [29, 31, 33–41] established an OP model by bilateral oophorectomy, two studies [27, 30] by intramuscular injection of DXM (2.5 mg/kg, biw) for 12 weeks, two studies [17, 28] by oral gavage of 7% tretinoin (10 ml/kg/d, qd) for 2 weeks, and 1 study [32] by oral gavage of prednisone (0.005 mg/kg/d, qd) for 14 weeks. Anesthetics were reported in ten studies [17, 27, 29, 32–34, 37–40], of which chloral hydrate was reported in five studies [27, 29, 32–34], pentobarbital sodium in four studies [17, 37–39], and ketamine in one study [40]. Detailed information of IFP in each study is displayed in Table 3. With regard to the specific IFP, isopsoralen was reported in five studies [27, 28, 32, 34, 36], psoralen in seven studies [17, 29–31, 35, 37, 40], bakuchiol in two studies [33, 41], psoralidin in one study [39], and bavachin in one study [38]. Fifteen studies [17, 27–40] used a dose gradient of IFP by oral administration ranging from 16 mg/kg/d to 1.26 g/kg/d, and one study [41] used subcutaneous injection administration by 30 mg/kg/d. In terms of primary outcome, BMD-femur was measured in twelve studies [27–32, 34, 37–41], BMD-lumbar spine in seven studies [27–29, 32, 34, 37, 39], S-OCN in five studies [28–30, 32, 40], BV/TV in five studies [27, 31, 33, 36, 37], Tb.N in six studies [17, 27, 31, 33, 36, 37], Tb.Th in four studies [17, 31, 33, 36], Tb.Sp in five studies [17, 31, 33, 36, 37], bone maximum load in six studies [30–32, 34, 35, 37], and elasticity modulus in six studies [30, 31, 34, 35, 37, 39].

### 3.3. Study Quality

The CAMARADES 10-item quality checklist was adopted to judge the risk of bias of each study, and the number of criteria met varied from 4/10 to 7/10 with the average of 5.25. Detailed results of methodological quality are presented in Table 4. All the included studies were peer-reviewed publications; three studies [31, 37, 39] did not mention control of temperature. Two [32, 34] of the included studies did not declare randomisation. The ways of blinding induction of model were reported in seven studies [29–31, 33, 35, 36, 41], and all of them reported that the animals were grouped randomly after modeling. No study mentioned the calculation of the sample size, and none used a blinding method during outcome assessment and an appropriate animal model. Ten studies [17, 27, 29, 32–34, 37–40] used the anesthetic without protective and toxic effects on bones. Compliance with animal welfare regulations was not described in four studies [33, 35, 37, 40], and the potential conflict of interests was not mentioned in four studies [30, 32, 35, 40].

### 3.4. Effectiveness

#### 3.4.1. S-OCN

**Figure 3:**
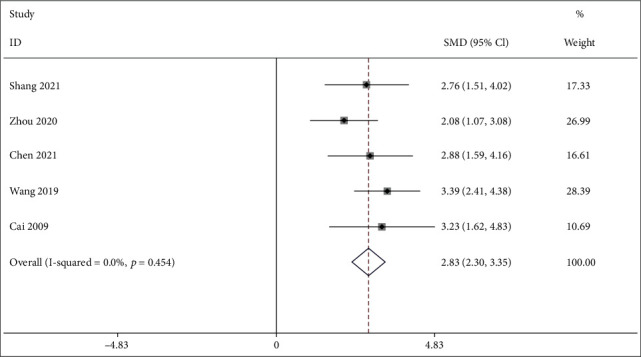
Forest plot of IFP versus control with regard to S-OCN.

Five studies [28–30, 32, 40] compared IFP with a control group regarding S-OCN. As shown in Figure 3, the pooled results showed that IFP significantly increased the S-OCN in contrast with control (SMD = 2.825; 95%CI = 2.302 to 3.349; *P* < 0.001; heterogeneity *χ*^2^ = 3.66, df = 4, *I*^2^ = 0%, *P* = 0.454, Figure 3).

#### 3.4.2. BMD

**Figure 4:**
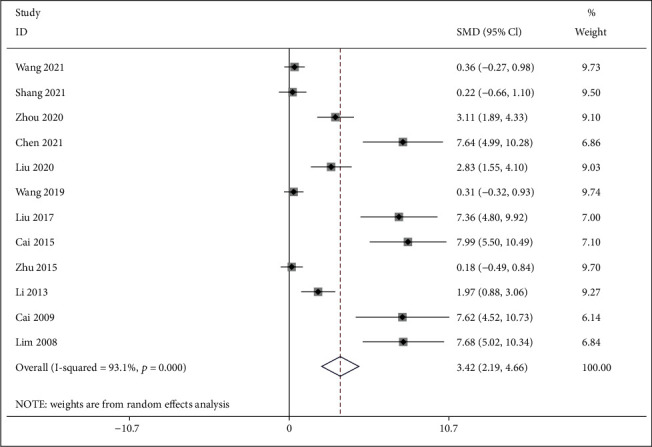
Forest plot of IFP versus control with regard to BMD-femur.

**Figure 5:**
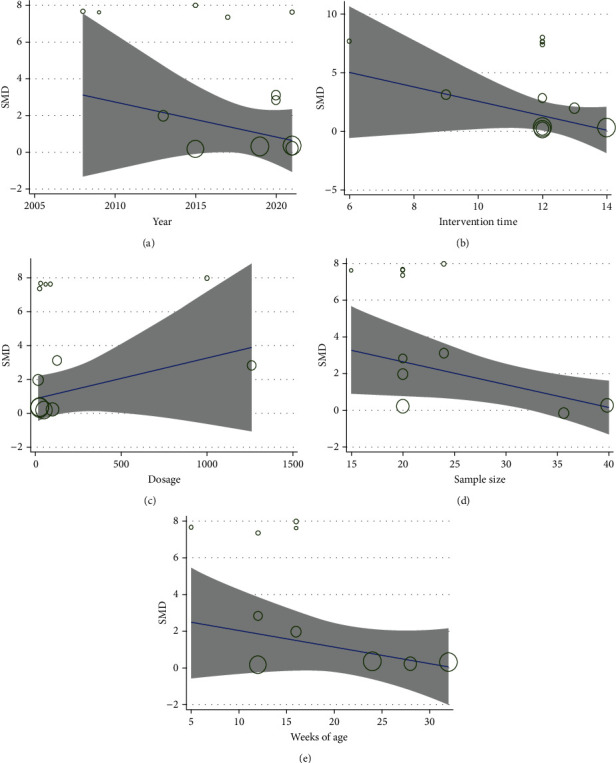
Metaregression analysis of BMD-femur: (a) publication year; (b) intervention time; (c) dosage of IFP; (d) sample size; (e) weeks of age.

*(1) BMD-Femur*. Twelve studies [27–32, 34, 37–41] reported IFP with the control group according to BMD at the femur. The pooled results indicated that IFP was significant for lifting BMD at the femur compared to the control group (SMD = 3.424; 95%CI = 2.186 to 4.661; *P* < 0.001, heterogeneity *χ*^2^ = 159.09, df = 11, *I*^2^ = 93.1%, *P* < 0.001, Figure 4). The causes of heterogeneity in the results were explored by metaregression. The metaregression analysis for sample size, intervention time, dosage of IFP, publication year, and age of animals (weeks) was performed to analyze the potential sources of interstudy heterogeneity (Figure 5). Overall, the sample size (*β* = −0.244; *P* = 0.028; Adj *R*^2^ = 35.83%) might be one of the sources of heterogeneity. However, intervention time (*β* = −0.644; *P* = 0.216; Adj *R*^2^ = 8.40%), publication year (*β* = 0.209; *P* = 0.122; Adj *R*^2^ = 15.95%), dosage of IFP (*β* = 0.001; *P* = 0.595; Adj *R*^2^ = −7.13%), and age of animals (*β* = 0.117; *P* = 0.062; Adj *R*^2^ = 31.60%) were not the major causes of heterogeneity for BMD at the femur.

**Figure 6:**
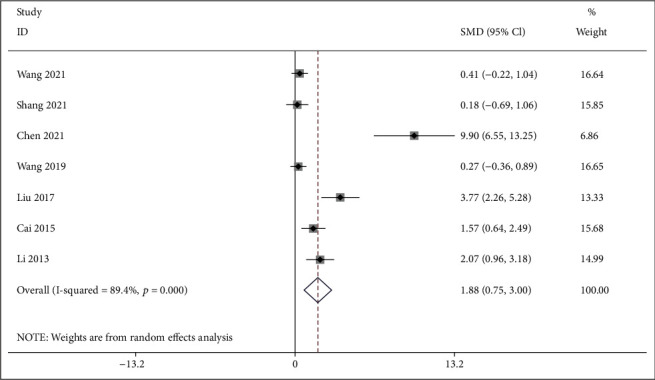
Forest plot of IFP versus control with regard to BMD-lumbar spine.

*(2) BMD-Lumbar Spine*. Seven studies [27–29, 32, 34, 37, 39] compared IFP with the control group about the BMD at the lumbar spine. As shown in Figure 6, the pooled results showed that IFP was significant for improving BMD at the lumbar spine compared with the control group (SMD = 1.880; 95%CI = 0.754 to 3.005; *P* = 0.001; heterogeneity *χ*^2^ = 56.71, df = 6, *I*^2^ = 89.4%, *P* < 0.001).

#### 3.4.3. BMD-Related Indicator under Micro-CT

**Figure 7:**
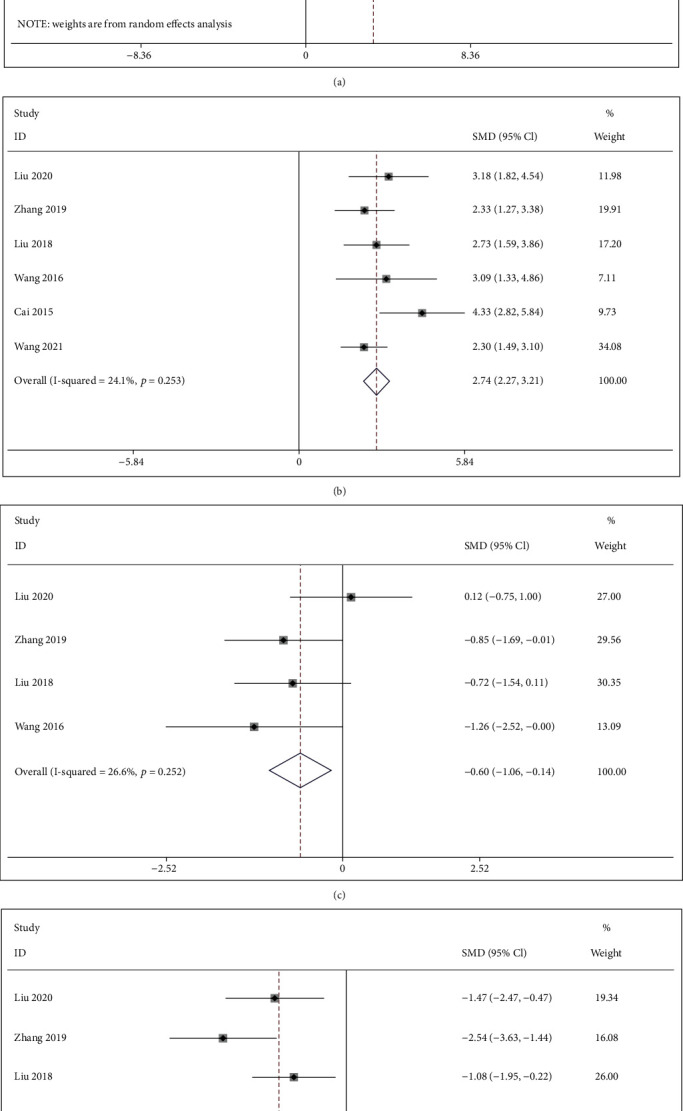
Forest plot of IFP versus control with regard to BMD-related indicator under micro-CT: (a) BV/TV; (b) Tb.N; (c) Tb.Th; (d) Tb.Sp.

*(1) BV/TV*. Five studies [27, 31, 33, 36, 37] reported IFP versus the control group according to BV/TV. The pooled results indicated that IFP was significant for raising BV/TV compared to the control group (SMD = 3.433; 95%CI = 1.412 to 5.455; *P* = 0.001; heterogeneity *χ*^2^ = 47.06, df = 4, *I*^2^ = 91.5%, *P* < 0.001, Figure 7(a)).

*(2) Tb.N*. There were six studies [17, 27, 31, 33, 36, 37] comparing IFP with the control group about Tb.N. The pooled results indicated that IFP significantly increased Tb.N compared to the control group (SMD = 2.737; 95%CI = 2.267 to 3.208; *P* < 0.001; heterogeneity *χ*^2^ = 6.59, df = 5, *I*^2^ = 24.1%, *P* = 0.253, Figure 7(b)).

*(3) Tb.Th*. Four studies [17, 31, 33, 36] compared IFP with the control group regarding the Tb.Th. As shown in Figure 7(c), the pooled results showed that IFP was significant for decreasing Tb.Th compared with the control group (SMD = −0.600; 95%CI = −1.056 to − 0.145; *P* = 0.010; heterogeneity *χ*^2^ = 4.09, df = 3, *I*^2^ = 26.6%, *P* = 0.252).

*(4) Tb.Sp*. Five studies [17, 31, 33, 36, 37] compared IFP with the control group with regard to Tb.Sp. The available data demonstrated that IFP significantly reduced Tb.Sp in contrast with the control group (SMD = −1.393; 95%CI = −1.833 to − 0.954; *P* < 0.001; heterogeneity *χ*^2^ = 5.60, df = 4, *I*^2^ = 28.6%, *P* = 0.231, Figure 7(d)).

#### 3.4.4. Bone Biomechanical Indicator

**Figure 8:**
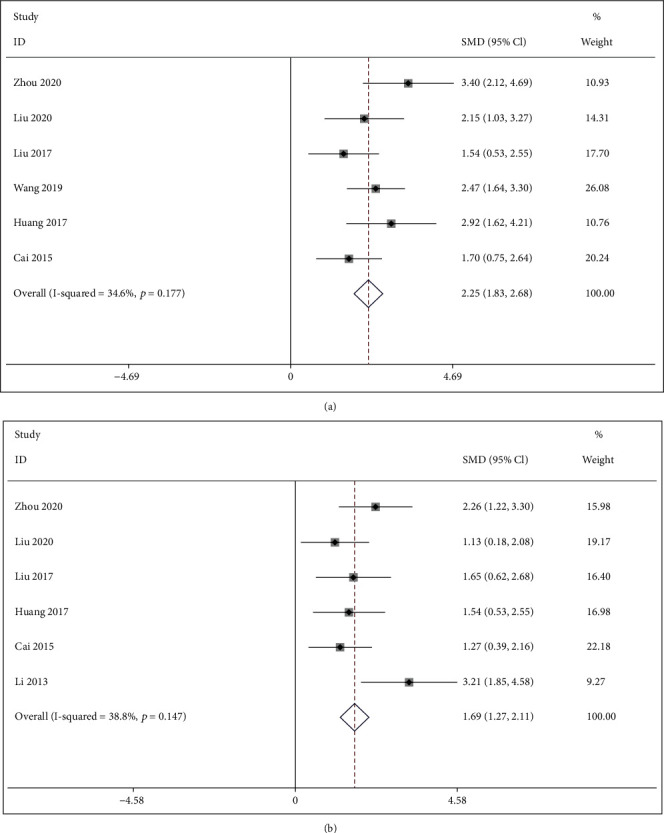
Forest plot of IFP versus control with regard to bone biomechanical indicator: (a) bone maximum load; (b) elasticity modulus.

*(1) Bone Maximum Load*. There were six studies [30–32, 34, 35, 37] comparing IFP with the control group about the bone maximum load. The pooled results indicated that IFP significantly improved bone maximum load compared to the control group (SMD = 2.253; 95%CI = 1.828 to 2.678; *P* < 0.001; heterogeneity *χ*^2^ = 7.65, df = 5, *I*^2^ = 34.6%, *P* = 0.177, Figure 8(a)).

*(2) Elasticity Modulus*. Six studies [30, 31, 34, 35, 37, 39] reported IFP with the control group according to elasticity modulus. The pooled results indicated that elasticity modulus in the IFP group was significantly larger than that in the control group (SMD = 1.691; 95%CI = 1.274 to 2.107; *P* < 0.001; heterogeneity *χ*^2^ = 8.18, df = 5, *I*^2^ = 38.8%, *P* = 0.147, Figure 8(b)).

### 3.5. Subgroup Analysis

**Figure 9:**
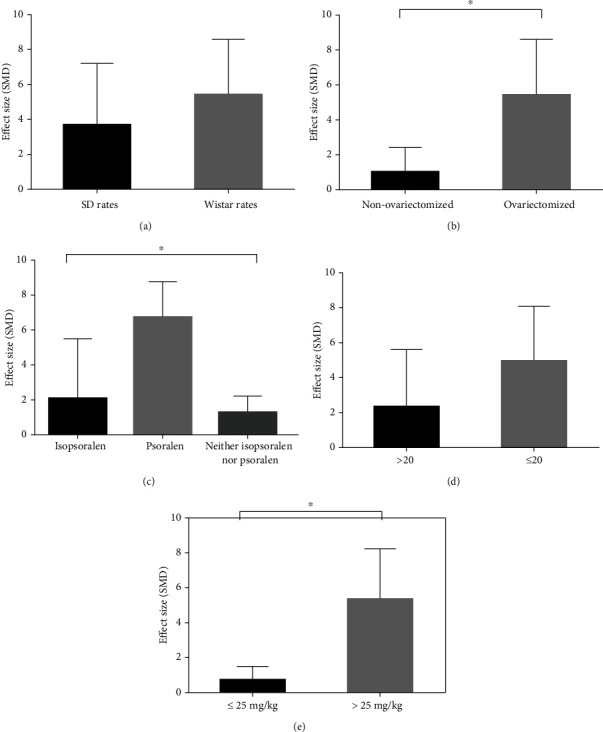
Subgroup analyses of the BMD-femur. (a) The different effect sizes between the SD rat group and the Wistar rat group. (b) The different effect sizes between the ovariectomized model group and the nonovariectomized model group. (c) The different effect sizes between the different kinds of IFP group. (d) The different effect sizes between different sample size groups. (e) The different effect sizes between different dosage groups. ^∗^*P* < 0.05 between subgroups.

**Figure 10:**
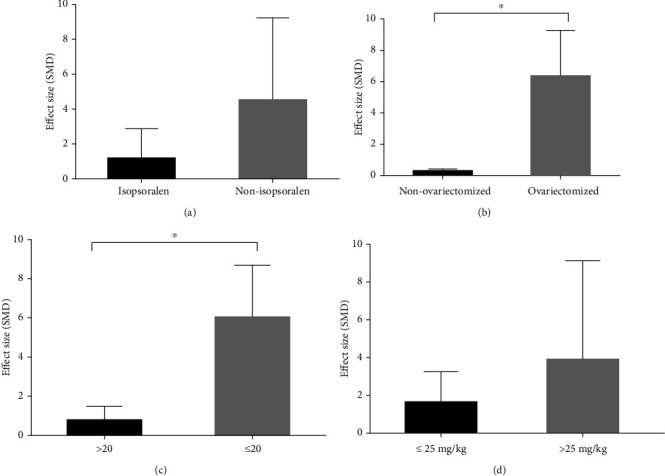
Subgroup analyses of the BMD-lumbar spine. (a) The different effect sizes between the different kinds of IFP groups. (b) The different effect sizes between the ovariectomized model group and the nonovariectomized model group. (c) The different effect sizes between different sample size groups. (d) The different effect sizes between different dosage groups. ^∗^*P* < 0.05 between subgroups.

The potential confounding factors (including animal species, modeling methods, kind of IFP, sample size, and dosages of IFP) that may increase the heterogeneity of outcome measures were explored using subgroup analysis of BMD-femur and BMD-lumbar spine. As for BMD-femur, the subgroup analysis of animal species showed that no difference was seen in the effect size between the SD rat group and the Wistar rat group (SMD = 3.654 ± 3.560 versus SMD = 5.368 ± 3.192, respectively, *P* = 0.544, Figure 9(a)), and heterogeneity of both groups did not decrease obviously. In the subgroup analysis of modeling methods, the ovariectomized model group showed better effect size than the nonovariectomized model group (SMD = 5.409 ± 3.193 versus SMD = 1.000 ± 1.408, respectively, *P* = 0.027, Figure 9(b)) with significantly reduced heterogeneity of both groups. In the subgroup analysis of kind of IFP, significant difference was found between the three groups (SMD = 2.063 ± 3.533 versus SMD = 6.839 ± 2.090 versus SMD = 1.276 ± 0.963, respectively, *P* = 0.021, Figure 9(c)), and the heterogeneity of the three groups decreased substantially. However, no difference was shown between the high-sample group (>20) and the low-sample group (≤20) (SMD = 2.390 ± 3.365 versus SMD = 5.047 ± 3.248, respectively, *P* = 0.199, Figure 9(d)). Besides, the high-dosage IFP group (>25 mg/kg, qd) showed greater effect size than in the low-dosage IFP (≤25 mg/kg, qd) (SMD = 5.535 ± 3.013 versus SMD = 0.750 ± 0.815, *P* = 0.0122, Figure 9(e)), and heterogeneity of two groups reduced substantially. In terms of BMD-lumbar spine, the subgroup analysis of kind of IFP showed that no significant difference was revealed between the isopsoralen group and nonisopsoralen group (SMD = 1.157 ± 1.743 versus SMD = 4.512 ± 4.672, respectively, *P* = 0.2499, Figure 10(a)) without a significant decline in heterogeneity between subgroups. In the subgroup analysis of modeling methods, the ovariectomized model group showed better effect size than the nonovariectomized model group (SMD = 6.326 ± 2.941 versus SMD = 0.2870 ± 0.1149, respectively, *P* = 0.0179, Figure 10(b)) with significantly reduced heterogeneity of both groups. Moreover, the low-sample group (≤20) exhibited better effect size than the high-sample group (>20) (SMD = 5.980 ± 2.690 versus SMD = 0.7480 ± 0.7130, respectively, *P* = 0.0254, Figure 10(c)), and the heterogeneity experienced a marked decline in the high-sample group, whereas no difference was found between the high-dosage IFP group (>25 mg/kg, qd) and the low-dosage IFP group (≤25 mg/kg, qd) (SMD = 3.883 ± 5.255 versus SMD = 1.629 ± 1.644, respectively, *P* = 0.539, Figure 10(d)) without a significant decrease in heterogeneity between subgroups.

### 3.6. Publication Bias and Sensitivity Analysis

**Figure 11:**
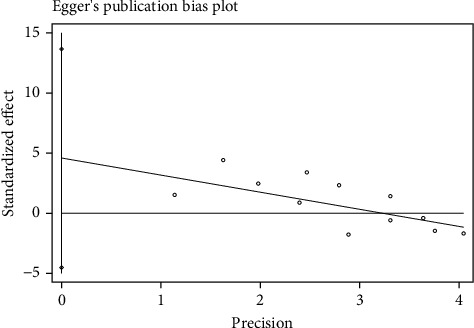
Egger's test of BMD-femur.

Egger's test (Figure 11) was used to assess the potential publication bias of the BMD-femur in this meta-analysis. The *P* values from Egger's tests indicated that there was no significant publication bias for BMD-femur (*P* = 0.416).

**Figure 12:**
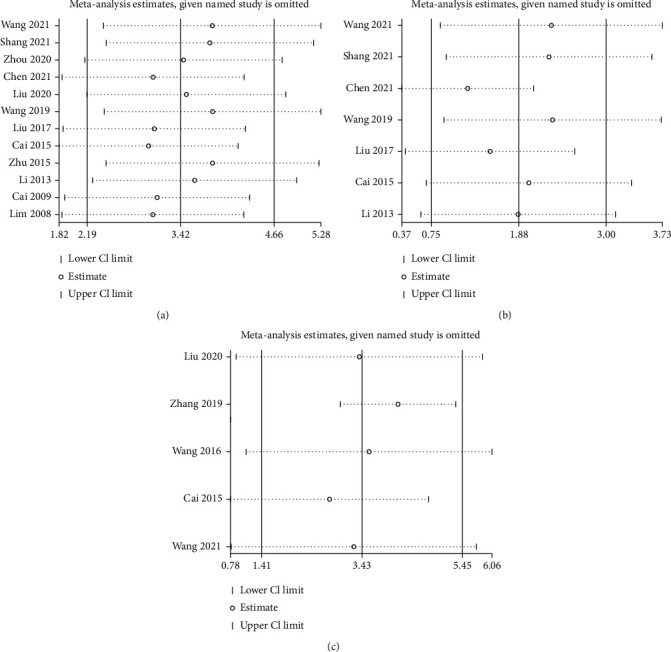
Sensitivity analysis for BMD-femur (a), BMD-lumbar spine (b), and BV/TV (c).

To determine the influence of each study on the pooled data for BMD-femur, BMD-lumbar spine, and BV/TV to verify the robustness of our results, sensitivity analysis was performed by omitting one study at a time and calculating the pooled data for the remaining studies. The results of the sensitivity analysis indicated that no significant effect was observed after excluding any single study, suggesting that the results of this meta-analysis were relatively robust (Figure 12).

### 3.7. GRADE Assessment

The GRADE system was used to assess the level of evidence for the outcomes, which indicated moderate, low, or very low quality with methodological problems and heterogeneity problem. The GRADE evidence profiles are shown in Table 5.

## 4. Discussion

### 4.1. Summary of Evidence

This is the first preclinical systematic review and meta-analysis to estimate the efficacy and possible mechanism of IFP for the OP animal model. Sixteen high-quality studies involving 379 animals with the OP model were enrolled in the analysis. The primary findings of present systematic review illustrated that IFP could significantly increase the S-OCN, BMD, BV/TV, and Tb.N while Tb.Sp and Tb.Th were remarkably decreased by IFP in OP model animals. Moreover, IFP could significantly improve the bone biomechanical indicator bone maximum load and elasticity modulus. Therefore, the findings revealed that FP is a potential anti-OP drug through multiple mechanisms. However, the outcomes BMD-femur, BMD-lumbar spine, and BV/TV represented high heterogeneity in our meta-analysis. According to the results of subgroup analysis and metaregression in our study, the source of heterogeneity was from the sample size, OP modeling methods, kind of IFP, and dosages of IFP. Thus, more high-quality studies involving large sample sizes should be conducted to confirm our findings.

### 4.2. Strengths

The strengths of this meta-analysis study consisted of a clearly defined research question, which reduced the bias in the choice of the included studies, fidelity, and consistency to a precise research approach that we designed before the meta-analysis, an in-depth search of the literature, the agreement between the two researchers regarding the entry data components, and the quality control appraisal of all the data. The quality of included original studies was relatively moderate, which suggests that the results were relatively reliable. Additionally, the number of trials and the overall sample size was comparatively large (16 trails with 379 animals). We performed subgroup assessments and metaregression evaluation to identify the origin of heterogeneity. Consequently, no publication bias was reported in this meta-analysis, and sensitivity estimation revealed that the findings of this meta-analysis are comparatively robust.

### 4.3. Limitations

Some limitations that may affect the accuracy of the study should be considered. Firstly, the included primary studies had some intrinsic and methodological shortcomings: (1) Only 14 trials had sufficient information on the generation of random allocation. (2) The blinding procedure and sample size calculation were not reported or remained unclear in some studies, making it a challenge to bias findings unintentionally or intentionally and to help allow the credibility of study conclusions. Secondly, selection bias was unavoidable because only eight frequently used databases were searched for English and Chinese language studies. Therefore, the potentially relevant studies published in other languages could have been left out. Thirdly, the absence of negative studies might have led to the true effect of IFP being overestimated. Fourthly, though the metaregression and subgroup analysis were done, the high heterogeneity of BMD-femur, BMD-lumbar spine, and BV/TV could not be neglected. The OP modeling methods, the specific kind of IFP, dosage of IFP, administration approaches, and period of IFP treatments differed remarkably in the included studies. This heterogeneousness could compromise the viability of our findings. Fifthly, most of the included studies in the meta-analysis were conducted in China, a potential limitation to the generalizability of our findings. Sixthly, the overall quality of evidence of this study was low (Table 5). Finally, many of the included studies suffer from significant sources of bias; this also will jeopardize the validity of results.

### 4.4. Implications

High-quality methodologies of studies are the cornerstones of translating animal research into clinical drug treatments for human disease [42]. The score (mean 5.25) by prudent assessment of included studies was better than that of most studies of traditional Chinese medicine [43]. There were limitations in terms of blinding and sample size calculation. The blinding methods in the animal model establishment and outcome assessment were usually seen as technical difficulties for most studies. A sample size calculation could avoid the waste of resources caused by oversize and the imprecision of study result by undersize; the specific steps could be referred from the literature [44]. Besides, the Animal Research: Reporting of In Vivo Experiments (ARRIVE) guidelines are aimed at improving the quality of research reports by guiding complete and transparent reporting of in vivo animal research. Employing of experimental animals with comorbidities such as advanced age, obesity, hyperglycemia, or other risk factors may be more in line with the physiology of OP patients and may be helpful for the clinical translation of experimental results. These should be adopted in the future study management of IFP for OP.

Using different animal models at different research stages of disease is crucial to study the pathophysiology and treatments [45]. Factors that need to be considered include pathogenesis of model, availability of the animals, technical requirements, and cost and ethical considerations [46]. According to the pathogenesis, animal models of OP can be divided into two types: models with increased bone resorption as the dominant mechanism (such as ovariectomized OP model, disused OP model, tretinoin induction OP model, nutritional OP model, and glucocorticoid OP model) and models with reduced bone formation as the dominant mechanism (such as senile OP model) [47]. This study comprehensively includes the ovariectomized OP model and nonovariectomized OP model (tretinoin induction OP model and glucocorticoid OP model) to evaluate the efficacy and mechanisms of IFP for OP. The results of subgroup analysis suggested that the ovariectomized OP model group showed better effect size than the nonovariectomized OP model group in regard to BMD-femur (SMD = 5.409 ± 3.193 versus SMD = 1.000 ± 1.408, respectively, *P* = 0.027, Figure 9(b)), which suggests that the different OP model methods may be the source of high heterogeneity. In clinical practice, postmenopausal OP has a similar pathogenesis with ovariectomized OP model [48]. The ovariectomized OP model has become the most widely used animal model to study OP [49], and it also was the most adopted model in eligible studies. Thus, we suggest an ovariectomized OP model be adopted to assess OP in future studies.

The results of subgroup analysis regarding the specific kind of IFP in BMD-femur demonstrated that the prosalen group gave a higher effect size than the isoprosalen group and neither the isoprosalen nor the prosalen group (SMD = 2.063 ± 3.533 versus SMD = 6.839 ± 2.090 versus SMD = 1.276 ± 0.963, respectively, *P* = 0.021, Figure 9(c)), indicating that different kinds of IFP might be the origin of high heterogeneity. Psoralen is one of the most major ingredients of FP and was also the most used IFP in the included studies (reported in 7 studies) because it has the highest content in FP and is easy to extract from FP. Besides, psoralen has been reported to possess potential anti-OP effects in animal OP models through multiple signal pathways [17, 29, 35]. For example, psoralen could significantly increase BMD of femur and vertebra, serum levels of calcium, osteocalcin, N-terminal propeptide of type 1 procollagen (P1NP), bone morphogenetic protein 2 (BMP2), and vascular endothelial growth factor (VEGF) via phosphatidylinositol 3-kinase/protein kinase B/mammalian target of rapamycin (PI3K/Akt/mTOR) in postmenopausal rats [29]. Isoprosalen is another furanocoumarin compound of FP and is derived from psoralen. Analogously, several studies have shown that isoprosalen exerted mighty protective effects of OP in animal OP models [27, 28, 36]. Isopsoralen could increase BMD and improve serological indicators inhibiting Axin2/peroxisome proliferator activated receptor (PPAR-*γ*) signaling pathway and activating Wnt signaling pathway in rats with glucocorticoid-induced OP [27]. Other IFP including bakuchiol, bavachin, and psoralidin were also reported in our included studies; however, the number of studies was relatively less and their effect size was lower than prosalen and isoprosalen. Therefore, prosalen and isoprosalen may be recommended as potential candidates of anti-OP drugs in the future studies. However, their safety and toxicity should be taken into consideration, which will be the direction of future research.

### 4.5. Possible Mechanisms

**Figure 13:**
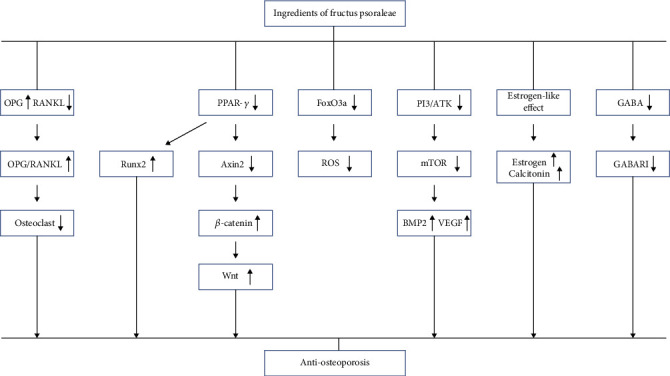
A schematic representation of antiosteoporosis mechanisms of IFP.

Systemic review of preclinical studies is conducive to understand comprehensively pathological mechanisms of disease and pharmacological effects of drugs [50]. The possible mechanisms of IFP that mediated anti-OP effects in the included studies are summed up as follows: (1) *Osteoprotegerin/receptor activator of nuclear factor-κB ligand/receptor activator of nuclear factor-κB (OPG/RANKL/RANK) signal pathway*: IFP could highly increase OPG secretion and reduce RANKL expression, resulting in an enhancement in OPG/RANKL ratio, which leads to a decrease in the number and activity of osteoclasts [30, 31, 34]. (2) *PPAR-γ/Axin2/Wnt signal pathway*: IFP suppressed the PPAR-*γ*/Axin2 signaling pathway involved in lipid metabolism, blocked the inhibition of Axin2 on the Wnt signaling pathway, upregulated the expression of *β*-catenin protein, and made *β*-catenin form a complex with nuclear transcription factors after entering the nucleus. Eventually, *β*-catenin activated the Wnt signaling pathway to regulate the lipid metabolism of rat bone marrow, thereby affecting bone metabolism and promoting bone formation [27]. Besides, the inhibition of PPAR-*γ* resulted in the increase of Runx2 expression, which facilitates osteogenic differentiation of bone mesenchymal stem cells in rats [36]. (3) *Antioxidative stress through the forkhead box O3a (FoxO3a)/Axin2/Wnt signal pathway*: IFP inhibited the activation of the FoxO3a signaling pathway through its antioxidant effect; meanwhile, it upregulated the expression of *β*-catenin, bound it to FoxO3a, initiated the transcriptional program that regulates bone tissue cell apoptosis and eliminates reactive oxygen species (ROS), which promotes the generation and formation of osteoblasts. Then, the expression of Axin2 was downregulated by removing excessive ROS, the PPAR-*γ*/Axin2 signaling pathway was inhibited to participate in lipid metabolism and the inhibition of Axin2 on the Wnt signaling pathway was blocked, which ultimately exerts its anti-OP effect [32]. (4) *PI3K/Akt/mTOR signaling pathway*: IFP restrained the expression of PI3K/Akt/mTOR in the rat's femur and increased the levels of BMP2 and VEGF, which improves bone formation and angiogenesis [29]. (5) *Estrogen-like effect*: IFP had the similar effect of phytoestrogen on inhibiting bone resorption by participating in the binding of estrogen receptor. On the one hand, IFP may promote the synthesis and secretion of estrogen outside the ovary [40, 41]. On the other hand, it may enhance the secretion of thyroid calcitonin to exert it anti-OP effect [39]. (6) *Gamma-aminobutyric acid/gamma-aminobutyric acid receptor (GABA/GABA_B_RI) signaling pathway*: IFP was observed to reduce the production of GABA and GABA_B_RI to inhibit the activity of osteoblast [38]. The mechanism diagram is summarized in Figure 13.

## 5. Conclusion

This preclinical systematic review provided preliminary evidence that IFP was capable of partially exerting anti-OP effects in animal models probably through the OPG/RANKL/RANK, PPAR-*γ*/Axin2/Wnt, antioxidative stress via FoxO3a/Axin2/Wnt, PI3K/Akt/mTOR, estrogen-like effect, and GABA/GABA_B_RI signaling pathway. Taken together, the findings suggest the possibility of developing IFP as a drug for the clinical treatment of OP.

## Figures and Tables

**Table 1:** PRISMA 2020 checklist.

Section and topic	Item #	Checklist item	Location where item is reported
*Title*			
Title	1	Identify the report as a systematic review	Page 1
*Abstract*			
Abstract	2	See the PRISMA 2020 for abstract checklist	Page 2
*Introduction*			
Rationale	3	Describe the rationale for the review in the context of existing knowledge	Page 4
Objectives	4	Provide an explicit statement of the objective(s) or question(s) the review addresses	Page 6
*Methods*			
Eligibility criteria	5	Specify the inclusion and exclusion criteria for the review and how studies were grouped for the syntheses	Page 7
Information sources	6	Specify all databases, registers, websites, organisations, reference lists, and other sources searched or consulted to identify studies. Specify the date when each source was last searched or consulted	Page 7
Search strategy	7	Present the full search strategies for all databases, registers, and websites, including any filters and limits used	Page 7
Selection process	8	Specify the methods used to decide whether a study met the inclusion criteria of the review, including how many reviewers screened each record and each report retrieved, whether they worked independently, and, if applicable, details of automation tools used in the process	Page 8
Data collection process	9	Specify the methods used to collect data from reports, including how many reviewers collected data from each report, whether they worked independently, any processes for obtaining or confirming data from study investigators, and, if applicable, details of automation tools used in the process	Page 9
Data items	10a	List and define all outcomes for which data were sought. Specify whether all results that were compatible with each outcome domain in each study were sought (e.g., for all measures, time points, analyses), and if not, the methods used to decide which results to collect	Page 9
	10b	List and define all other variables for which data were sought (e.g., participant and intervention characteristics, and funding sources). Describe any assumptions made about any missing or unclear information	Page 9
Study risk of bias assessment	11	Specify the methods used to assess risk of bias in the included studies, including details of the tool(s) used, how many reviewers assessed each study and whether they worked independently, and, if applicable, details of automation tools used in the process	Page 9
Effect measures	12	Specify for each outcome the effect measure(s) (e.g., risk ratio, mean difference) used in the synthesis or presentation of results	Page 10
Synthesis methods	13a	Describe the processes used to decide which studies were eligible for each synthesis (e.g., tabulating the study intervention characteristics and comparing against the planned groups for each synthesis (item #5))	Page 10
	13b	Describe any methods required to prepare the data for presentation or synthesis, such as handling of missing summary statistics or data conversions	Page 10
	13c	Describe any methods used to tabulate or visually display results of individual studies and syntheses	Page 10
	13d	Describe any methods used to synthesize results and provide a rationale for the choice(s). If meta-analysis was performed, describe the model(s), method(s) to identify the presence and extent of statistical heterogeneity, and software package(s) used	Page 10
	13e	Describe any methods used to explore possible causes of heterogeneity among study results (e.g., subgroup analysis, meta-regression)	Page 10
	13f	Describe any sensitivity analyses conducted to assess robustness of the synthesized results	Page 10
Reporting bias assessment	14	Describe any methods used to assess risk of bias due to missing results in a synthesis (arising from reporting biases)	Page 10
Certainty assessment	15	Describe any methods used to assess certainty (or confidence) in the body of evidence for an outcome	Page 10
*Results*			
Study selection	16a	Describe the results of the search and selection process, from the number of records identified in the search to the number of studies included in the review, ideally using a flow diagram	Page 10
	16b	Cite studies that might appear to meet the inclusion criteria, but which were excluded, and explain why they were excluded	Page 10
Study characteristics	17	Cite each included study and present its characteristics	Page 11
Risk of bias in studies	18	Present assessments of risk of bias for each included study	Page 12
Results of individual studies	19	For all outcomes, present, for each study, the following: (a) summary statistics for each group (where appropriate) and (b) an effect estimate and its precision (e.g., confidence/credible interval), ideally using structured tables or plots	Table1
Results of syntheses	20a	For each synthesis, briefly summarize the characteristics and risk of bias among contributing studies	Pages 13-15
	20b	Present results of all statistical syntheses conducted. If meta-analysis was done, present for each the summary estimate and its precision (e.g., confidence/credible interval) and measures of statistical heterogeneity. If comparing groups, describe the direction of the effect	Pages 13-15
	20c	Present results of all investigations of possible causes of heterogeneity among study results	Pages 15-17
	20d	Present results of all sensitivity analyses conducted to assess the robustness of the synthesized results	Page 17
Reporting biases	21	Present assessments of risk of bias due to missing results (arising from reporting biases) for each synthesis assessed	Page 17
Certainty of evidence	22	Present assessments of certainty (or confidence) in the body of evidence for each outcome assessed	Page 17
*Discussion*			
Discussion	23a	Provide a general interpretation of the results in the context of other evidence	Page 18
	23b	Discuss any limitations of the evidence included in the review	Page 19
	23c	Discuss any limitations of the review processes used	Page 19
	23d	Discuss implications of the results for practice, policy, and future research	Pages 20-22
*Other information*			
Registration and protocol	24a	Provide registration information for the review, including register name and registration number, or state that the review was not registered	Page 7
	24b	Indicate where the review protocol can be accessed or state that a protocol was not prepared	Page 7
	24c	Describe and explain any amendments to information provided at registration or in the protocol	None
Support	25	Describe sources of financial or nonfinancial support for the review and the role of the funders or sponsors in the review	Page 25
Competing interests	26	Declare any competing interests of review authors	Page 25
Availability of data, code, and other materials	27	Report which of the following are publicly available and where they can be found: template data collection forms; data extracted from included studies; data used for all analyses; analytic code; any other materials used in the review	Page 24

*From:* Page MJ, McKenzie JE, Bossuyt PM, Boutron I, Hoffmann TC, Mulrow CD, et al. The PRISMA 2020 statement: an updated guideline for reporting systematic reviews. BMJ 2021; 372: n71. doi: 10.1136/bmj.n71. For more information, visit: http://www.prisma-statement.org/.

**Table 2:** Characteristics of the included studies.

Study (year)	Species (sex, *n* = experimental/control group, age)	Weight	Model (method)	Anesthetic	Experimental group	Control group	Outcome index	Intergroup differences
Chai et al. (2018) [21]	Female SD rats (20/20, 6 months old)	NG	By intramuscular injection of DXM (2.5 mg/kg, biw) for 12 weeks	Chloral hydrate	By oral gavage of ISO (25 mg/kg/d, qd) after modeling lasted 12 weeks	By oral gavage of isometric NS after modeling lasted 12 weeks	(1) BMD (F-BMD; L-BMD; P-BMD; total-BMD) (2) Tb.N, BV/TV (3) Serum ALP, TRACP, Ca, P (4) PPAR-*γ*, Axin2, *β*-catenin protein expression	(1) *P* < 0.05 (2) *P* < 0.05 (3) *P* < 0.05 (4) *P* < 0.05
van Luijk et al. (2013) [22]	Female SD rats (9/9, 7 weeks old)	180-220 g	By oral gavage of tretinoin (70 mg/kg/d, qd) for 2 weeks	NG	By oral gavage of ISO (100 mg/kg/d, qd) after modeling and lasted 12 weeks	By oral gavage of isometric NS after modeling and lasted 12 weeks	(1) BMD (F-BMD; L-BMD) (2) Serum Ca (3) Serum P (4) OCN (5) Runx2, MMP 13 mRNA, and protein expression	(1) *P* < 0.05 (2) *P* < 0.05 (3) *P* > 0.05 (4) *P* < 0.05 (5) *P* < 0.05
Moher et al. (2009) [23]	Female SD rats (10/10, NG)	200-280 g	Bilateral oophorectomy was performed on rats	Chloral hydrate	By oral gavage of PSO (88 mg/kg/d, qd) after modeling and lasted 12 weeks	By oral gavage of isometric NS after modeling and lasted 12 weeks	(1) BMD (F-BMD; L-BMD) (2) Serum Ca, PINP, OCN (3) Serum BMP2, VEGF (4) PI3K, AKT, mTOR mRNA, and protein expression	(1) *P* < 0.05 (2) *P* < 0.05 (3) *P* < 0.05 (4) *P* < 0.05
Macleod et al. (2004) [24]	Female and male Wistar rats (12/12, NG)	160-200 g	By intramuscular injection of DXM (2.5 mg/kg, biw) for 12 weeks	NG	By oral gavage of PSO (126 mg/kg/d, qd) after modeling and lasted 9 weeks	By oral gavage of isometric NS after modeling and lasted 9 weeks	(1) BMD (F-BMD) (2) Elasticity modulus, maximum load, yield load (3) Content of bone mineral salt (4) RANKL, OPG protein expression	(1) *P* < 0.05 (2) *P* < 0.05 (3) *P* < 0.05 (4) *P* < 0.05
Guyatt et al. (2011) [25]	Female SD rats (10/10, 3 months old)	NG	Bilateral oophorectomy was performed on rats	NG	By oral gavage of PSO (1.26 g/kg/d, qd) after modeling and lasted 12 weeks	By oral gavage of isometric NS after modeling and lasted 12 weeks	(1) BMD (F-BMD) (2) BV/TV, Tb.Th, Tb.N, Tb.Sp (3) Elasticity modulus, maximum load (4) Serum PINP, CTX, ALP (5) Serum AST, ALT (6) Runx2, OPG, RANKL, collagen-I protein expression	(1) *P* < 0.05 (2) *P* > 0.05 (3) *P* < 0.05 (4) *P* < 0.05 (5) *P* > 0.05 (6) *P* < 0.05
Balshem et al. (2011) [26]	Female SD rats (20/20, 8 months old)	200-220 g	By oral gavage of prednisone (0.005 mg/kg/d, qd) for 14 weeks	Chloral hydrate	By oral gavage of ISO (25 mg/kg/d, qd) after modeling and lasted 14 weeks	By oral gavage of isometric NS after modeling and lasted 14 weeks	(1) BMD (F-BMD; L-BMD; P-BMD; total-BMD) (2) Serum BAP, OCN, OPG, RANKL, TRACP5b (3) CAT, SOD, MDA (4) Elasticity modulus, bending energy, maximum load, stiffness coefficient (5) FoxO3a, Axin2, *β*-catenin protein expression	(1) *P* < 0.05 (2) *P* < 0.05 (3) *P* < 0.05 (4) *P* < 0.05 (5) *P* < 0.05
Wang et al. (2021) [27]	Female ICR mice (12/11, 6 weeks old)	20-22 g	Bilateral oophorectomy was performed on rats	Chloral hydrate	By oral gavage of bakuchiol (20 mg/kg/d, qd) after modeling and lasted 8 weeks	By oral gavage of isometric NS after modeling and lasted 8 weeks	(1) BV/TV, Tb.Th (2) Tb.N, Tb.Sp, DA (3) Maximum load (4) Serum OCN (5) Serum ALP, TRACP	(1) *P* > 0.05 (2) *P* < 0.05 (3) *P* < 0.05 (4) *P* < 0.05 (5) *P* > 0.05
Heinrich et al. (2020) [14]	Female and male Wistar rats (12/12, NG)	190-210 g	By oral gavage of tretinoin (70 mg/kg/d, qd) for 2 weeks	Pentobarbital sodium	By oral gavage of PSO (0.9 g/kg/d, qd) after modeling and lasted 8 weeks	By oral gavage of isometric NS after modeling and lasted 8 weeks	(1) Of BV, Conn.D (2) Tb.N, Tb.Sp (3) Tb.Th (4) Serum ACP (5) Serum ALP, ALP/ACP	(1) *P* < 0.05 (2) *P* < 0.05 (3) *P* > 0.05 (4) *P* < 0.05 (5) *P* > 0.05
Shang et al. (2021) [28]	Female SD rats (10/10, 3 months old)	210-230 g	Bilateral oophorectomy was performed on rats	Chloral hydrate	By oral gavage of ISO (25 mg/kg/d, qd) after modeling and lasted 12 weeks	By oral gavage of isometric NS after modeling and lasted 12 weeks	(1) BMD (F-BMD; L-BMD; total-BMD) (2) Elasticity modulus, bending energy, maximum load (3) Serum OCN, TRACP 5b (4) OPG, RANKL protein expression	(1) *P* < 0.05 (2) *P* < 0.05 (3) *P* < 0.05 (4) *P* < 0.05
Chen and Tang (2021) [29]	Male C57BL/6 mice (10/10, 9 weeks old)	17.7 ± 1.1 g	Bilateral oophorectomy was performed on rats	NG	By oral gavage of PSO (20 mg/kg/d, qd) after modeling and lasted 6 weeks	By oral gavage of isometric NS after modeling and lasted 6 weeks	(1) Serum ALP, CTX (2) Elasticity modulus, maximum load	(1) *P* < 0.05 (2) *P* < 0.05
Zhou et al. (2020) [30]	Male C57BL/6 mice (6/6, NG)	NG	Bilateral oophorectomy was performed on rats	NG	By oral gavage of ISO (20 mg/kg/d, qd) after modeling and lasted 8 weeks	By oral gavage of isometric NS after modeling and lasted 8 weeks	(1) BV/TV, Tb.Th, Tb.N, Tb.Sp (2) Runx2, PPAR-*γ* protein expression	(1) *P* < 0.05 (2) *P* < 0.05
Liu et al. (2020) [31]	Female SD rats (12/12, 4 months old)	250 ± 25 g	Bilateral oophorectomy was performed on rats	Pentobarbital sodium	By oral gavage of PSO (1 g/kg/d, qd) after modeling and lasted 12 weeks	By oral gavage of isometric NS after modeling and lasted 12 weeks	(1) BMD (F-BMD; L-BMD) (2) BV/TV, Tb.Th, Tb.N, Tb.Sp (3) Elasticity modulus, maximum load (4) Serum ALP, IL-1, IL-6	(1) *P* < 0.05 (2) *P* < 0.05 (3) *P* < 0.05 (4) *P* < 0.05
Wang et al. (2019) [32]	Female SD rats (18/17, 3 months old)	180 ± 20 g	Bilateral oophorectomy was performed on rats	Pentobarbital sodium	By oral gavage of bavachin (50 mg/kg/d, qd) after modeling and lasted 12 weeks	By oral gavage of isometric NS after modeling and lasted 12 weeks	(1) BMD (F-BMD) (2) Urinary Ca (3) GABA, GABA_B_RI	(1) *P* < 0.05 (2) *P* < 0.05 (3) *P* < 0.05
Zhang et al. (2019) [33]	Female SD rats (12/12, 4 months old)	230 g	Bilateral oophorectomy was performed on rats	Pentobarbital sodium	By oral gavage of psoralidin (16 mg/kg/d, qd) after modeling and lasted 13 weeks	By oral gavage of isometric vegetable oil after modeling and lasted 13 weeks	(1) BMD (F-BMD; L-BMD) (2) Serum estradiol, calcitonin (3) Elasticity modulus	(1) *P* < 0.05 (2) *P* < 0.05 (3) *P* < 0.05
Liu et al. (2017) [34]	Female Wistar rats (8/7, 4 months old)	290.9 ± 1.5 g	Bilateral oophorectomy was performed on rats	Ketamine	By oral gavage of PSO (60 mg/kg/d, qd) after modeling and lasted 12 weeks	By oral gavage of isometric NS after modeling and lasted 12 weeks	(1) BMD (F-BMD) (2) Serum estradiol (3) Serum OCN, 1,25-dihydroxyvitamin D3, TNF-*α*	(1) *P* < 0.05 (2) *P* > 0.05 (3) *P* < 0.05
Huang and Zhou (2017) [35]	Female SD rats (10/10, 5 weeks old)	NG	Bilateral oophorectomy was performed on rats	NG	By subcutaneous injection of bakuchiol (30 mg/kg/d, qd) after modeling and lasted 6 weeks	By oral subcutaneous injection of isometric NS after modeling and lasted 6 weeks	(1) BMD (F-BMD) (2) Serum ALP, P, Ca, estradiol	(1) *P* < 0.05 (2) *P* < 0.05

NG: not given; SD: Sprague-Dawley; DXM: dexamethasone; ISO: isopsoralen; PSO: psoralen; NS: normal saline; BMD: bone mineral density; F-BMD: femoral bone mineral density; L-BMD: lumbar bone mineral density; P-BMD: pelvis bone mineral density: BV/TV: bone volume over total volume; Tb.N: trabecular linear density; Tb.Th: trabecular thickness; Tb.Sp: trabecular separation; ALP: alkaline phosphatase; ACP: acid phosphatase; AST: aspartate aminotransferase; ALT: alanine aminotransferase; TRACP: tartrate-resistant acid phosphatase; Ca: calcium; P: phosphorus; PPAR-*γ*: peroxisome proliferator activated receptor *γ*; OCN: osteocalcin; Runx2: runt-related transcription factor 2; MMP 13: matrix metalloproteinase 13; PINP: N-terminal propeptide of type 1 procollagen; BMP2: bone morphogenetic protein 2; VEGF: vascular endothelial growth factor; PI3K: phosphatidylinositol 3-kinase; AKT: protein kinase B; mTOR: mammalian target of rapamycin; RANKL: receptor activator of nuclear factor-*κ*B ligand; OPG: osteoprotegerin; CTX: C-terminal cross-linked telopeptide of type I collagen; CAT: catalase; SOD: superoxide dismutase; MDA: malondialdehyde; FoxO3a: forkhead box O3a; Conn.D: connectivity density; IL-1: interleukin-1; IL-6: interleukin-6; GABA: gamma-aminobutyric acid; GABA_B_RI: gamma-aminobutyric acid receptor; qd: once a day; biw: twice a week.

**Table 3:** Detailed information of IFP in each study.

Study (year)	Chemical composition	Source	Purity (%)	Quality control reported
Chai et al. (2018) [21]	Isopsoralen	Chengdu Ruisifen Biological Technology Co., Ltd., CHN	NG	Batch number: 20180122
van Luijk et al. (2013) [22]	Isopsoralen	Shanghai Chunyou Biotechnology Co., Ltd., CHN	≥98%	HPLC
Moher et al. (2009) [23]	Psoralen	Guangdong Jingxin Biological Technology Co., Ltd., CHN	98%	Batch number: 1202133231
Macleod et al. (2004) [24]	Psoralen	Xinjiang Qikang Harbowei Pharmaceutical Co., Ltd., CHN	≥98%	Batch number: 20151027
Guyatt et al. (2011) [25]	Psoralen	National Institute for Food and Drug Control	NG	HPLC
Balshem et al. (2011) [26]	Isopsoralen	Chengdu Ruisifen Biological Technology Co., Ltd., CHN	NG	Batch number: 20180122
Wang et al. (2021) [27]	Bakuchiol	Tianjin Crescent Lake Technology Co. Ltd., CHN	≥95%	HPLC
Heinrich et al. (2020) [14]	Psoralen	Shenzhen China Resources Sanjiu Pharmaceutical Co., Ltd., CHN	≥98%	Batch number: 20120920
Shang et al. (2021) [28]	Isopsoralen	National Institute for the Control of Pharmaceutical and Biological Products	≥98%	Batch number: 110738-201509
Chen and Tang (2021) [29]	Psoralen	Jiangsu Yongjian Pharmaceutical Co., Ltd., CHN	NG	NG
Zhou et al. (2020) [30]	Isopsoralen	Sigma-Aldrich Corporation, USA	≥99%	HPLC
Liu et al. (2020) [31]	Psoralen	Henan Luoyang Orthopedic Hospital, CHN	≥98%	HPLC
Wang et al. (2019) [32]	Bavachin	NG	NG	NG
Zhang et al. (2019) [33]	Psoralidin	School of Pharmacy of Central South University, CHN	≥98%	HPLC
Liu et al. (2017) [34]	Psoralen	The Second People's Hospital of Shanxi Province, CHN	NG	NG
Huang and Zhou (2017) [35]	Bakuchiol	Korea Research Institute of Chemical Technology	≥98%	HPLC

HPLC: high-performance liquid chromatography; NG: not given.

**Table 4:** Risk of bias of the included studies.

Study	A	B	C	D	E	F	G	H	I	J	Total
Chai et al. (2018) [21]	√	√	√			√			√	√	6
van Luijk et al. (2013) [22]	√	√	√						√	√	5
Moher et al. (2009) [23]	√	√	√	√		√			√	√	7
Macleod et al. (2004) [24]	√	√	√	√					√		5
Guyatt et al. (2011) [25]	√		√	√					√	√	5
Balshem et al. (2011) [26]	√	√				√			√		4
Wang et al. (2021) [27]	√	√	√	√		√				√	6
Heinrich et al. (2020) [14]	√	√	√			√			√	√	6
Shang et al. (2021) [28]	√	√				√			√	√	5
Chen and Tang (2021) [29]	√	√	√	√							4
Zhou et al. (2020) [30]	√	√	√	√					√	√	6
Liu et al. (2020) [31]	√		√			√				√	4
Wang et al. (2019) [32]	√	√	√			√			√	√	6
Zhang et al. (2019) [33]	√		√			√			√	√	5
Liu et al. (2017) [34]	√	√	√			√					4
Huang and Zhou (2017) [35]	√	√	√	√					√	√	6

Note: studies fulfilling the criteria of the following: A: peer-reviewed publication; B: control of temperature; C: random allocation to treatment or control; D: blinded induction of model (group randomly after modeling); E: blinded assessment of outcome; F: use of anesthetic without significant protective and toxic effects on bones; G: appropriate animal model (aged, hyperlipidemia, hypertensive, or diabetes); H: sample size calculation; I: compliance with animal welfare regulations (including three or more of the following points: preoperative anesthesia, postoperative analgesia, nutrition, disinfection, environment temperature, environment humidity, circadian rhythm, and euthanasia); J: statement of potential conflict of interests.

**Table 5:** GRADE evidence profile.

Quality assessment							No. of patients		Effect		Quality	Importance
No. of studies	Design	Risk of bias	Inconsistency	Indirectness	Imprecision	Other considerations	Ingredients of Fructus Psoraleae	Control	Relative (95% CI)	Absolute		
*S-OCN (better indicated by lower values)*												
5	Randomised trials	Serious^1^	No serious inconsistency	No serious indirectness	No serious imprecision	None	60	59	—	SMD 2.73 higher (2.2 to 3.25 higher)	⊕⊕⊕O Moderate	Important
*BMD-femur (better indicated by lower values)*												
12	Randomised trials	Serious^1^	Serious^2^	No serious indirectness	No serious imprecision	None	150	148	—	SMD 3.22 higher (2.04 to 4.41 higher)	⊕⊕OO Low	Critical
*BMD-lumbar spine (better indicated by lower values)*												
7	Randomised trials	Serious^1^	Serious^2^	No serious indirectness	No serious imprecision	None	92	92	—	SMD 1.76 higher (0.69 to 2.83 higher)	⊕⊕OO Low	Critical
*BV/TV (better indicated by lower values)*												
5	Randomised trials	Serious^1^	Serious^2^	No serious indirectness	No serious imprecision	None	60	60	—	SMD 3.29 higher (1.32 to 5.26 higher)	⊕⊕OO Low	Important
*Tb.N (better indicated by lower values)*												
6	Randomised trials	Very serious^1,3^	No serious inconsistency	No serious indirectness	No serious imprecision	None	72	72	—	SMD 2.64 higher (2.17 to 3.11 higher)	⊕⊕OO Low	Important
*Tb.Th (better indicated by lower values)*												
4	Randomised trials	Serious^1,4^	No serious inconsistency	No serious indirectness	No serious imprecision	None	40	40	—	SMD 0.57 lower (1.03 to 0.12 lower)	⊕⊕⊕O Moderate	Important
*Tb.Sp (better indicated by lower values)*												
5	Randomised trials	Serious^1^	No serious inconsistency	No serious indirectness	No serious imprecision	None	52	52	—	SMD 1.34 lower (1.78 to 0.89 lower)	⊕⊕⊕O Moderate	Important
*Bone maximum load (better indicated by lower values)*												
6	Randomised trials	Very serious^1,4^	No serious inconsistency	No serious indirectness	No serious imprecision	None	74	74	—	SMD 2.18 higher (1.75 to 2.6 higher)	⊕⊕OO Low	Critical
*Elasticity modulus (better indicated by lower values)*												
6	Randomised trials	Serious^1^	No serious inconsistency	No serious indirectness	No serious imprecision	None	64	64	—	SMD 1.62 higher (1.2 to 2.04 higher)	⊕⊕⊕O Moderate	Critical

GRADE working group grades of evidence: high quality: further research is very unlikely to change our confidence in the estimate of effect; moderate quality: further research is likely to have an important impact on our confidence in the estimate of effect and may change the estimate; low quality: further research is very likely to have an important impact on our confidence in the estimate of effect and is likely to change the estimate; very low quality: we are very uncertain about the estimate. ^1^Some of the included studies did not report the implementation of blinding. ^2^Heterogeneity (*I*^2^ > 50%, *P* < 0.05) was found. ^3^No details of random protocol were reported. ^4^Some of the included studies lack allocation concealment.

## Data Availability

Previously reported data were used to support this study. These prior studies and datasets are cited at relevant places within the text as references.

## Abbreviations

OP: Osteoporosis
FP: *Fructus Psoraleae*
IFP: Ingredients of *Fructus Psoraleae*
SD: Sprague-Dawley
DXM: Dexamethasone
ISO: Isopsoralen
PSO: Psoralen
NS: Normal saline
BMD: Bone mineral density
F-BMD: Femoral bone mineral density
L-BMD: Lumbar bone mineral density
BV/TV: Bone volume over total volume
Tb.N: Trabecular linear density
Tb.Th: Trabecular thickness
Tb.Sp: Trabecular separation
PPAR-*γ*: Peroxisome proliferator activated receptor *γ*
OCN: Osteocalcin
Runx2: Runt-related transcription factor 2
BMP2: Bone morphogenetic protein 2
VEGF: Vascular endothelial growth factor
PI3K: Phosphatidylinositol 3-kinase
AKT: Protein kinase B
mTOR: Mammalian target of rapamycin
RANKL: Receptor activator of nuclear factor-*κ* B ligand
OPG: Osteoprotegerin
FoxO3a: Forkhead box O3a
ROS: Reactive oxygen species
GABA: Gamma-aminobutyric acid
GABA_B_RI: Gamma-aminobutyric acid receptor.
